# Esports Associations and the Pursuit of Legitimacy: Evidence From Germany

**DOI:** 10.3389/fspor.2022.869151

**Published:** 2022-06-28

**Authors:** Heiko Heidenreich, Christian Brandt, Geoff Dickson, Markus Kurscheidt

**Affiliations:** ^1^Department of Sport Governance and Event Management, BaySpo - Bayreuth Center of Sport Science, University of Bayreuth, Bayreuth, Germany; ^2^Department of Management and Marketing, La Trobe University, Melbourne, VIC, Australia

**Keywords:** electronic gaming, sports governance, sports organization, institutional theory, conformance, manipulation, partial legitimacy

## Abstract

The dominant position of esports game publishers is a fundamental difference between the systemic governance of esports and traditional sports. There are no such equivalent organizations in traditional sports. As for-profit corporations, the publishers develop and market the electronic games as their commercial products and thus, possess exclusive property rights. Publishers control the virtual sporting environment and the rules of the game. In conventional sports, by contrast, non-profit associations administer their sports with the core task of developing the sport by regulations, playing rules, and licensing. There are, however, esports associations which resemble traditional leagues and national governing bodies. Given this, we explore how esports associations pursue legitimacy. This study is empirically motivated by the recent emergence of two esports associations in the insightful case of Germany and examines the pursuit of legitimacy by the World Esports Association (WESA) and the eSport-Bund Deutschland e.V. (ESBD). The study is based on a content analysis of 55 documents and nine interviews with relevant stakeholders. The findings show that the esports associations rely on conformance and manipulation strategies by transferring existing structures from traditional sports to esports. The most effective practices are lobbying for social and public acceptance of esports and creating supportive networks for esports development. While publishers possess an undisputed and taken-for-granted legitimacy based on their product property rights, esports associations struggle for recognition and acceptance. They may still have a long way to go, given that established associations in conventional sports have a history for decades. Yet, esports associations need to accept publisher dominance. Thus, they can only claim *partial legitimacy* within the esports ecosystem by targeting segments of stakeholders. Management, policy and theoretical implications of this key insight are finally presented.

## Introduction

Since the end of the 19th century, sports have been governed by an independent and non-profit network of international and continental federations, national sports organizations, as well as local, regional/provincial sports organizations, and both amateur and professional clubs and their associated leagues. The international federations, as apex organizations, are responsible for establishing rules and the format of international competitions (Chappelet, 2010) and are (almost always) undisputed as the legitimate governing bodies for their respective sports (Croci and Forster, 2004). However, this pyramidal structure cannot be reconciled with electronic sports (esports) because of the dominant position of corporate game publishers and developers.^1^ The key component of esports—the video game—depends on digital operating systems developed by these economic enterprises (Funk et al., 2018). Peng et al. (2020) consider game publishers the essential key stakeholder in the esports ecosystem. In most cases, they are developers, publishers, and, notably, the exclusive owners of the decisive property rights (Karhulahti, 2017; Abanazir, 2018; Peng et al., 2020). As commercial enterprises, game developers primarily pursue profit-oriented intentions (Abanazir, 2018; Funk et al., 2018).

This is somewhat ironic given that many of today's most successful esports games have their origin in non-commercial modifications of existing games designed and coded by enthusiastic gamers. Thus, the concept of community has a concise self-understanding in esports (Ashton, 2019; Xue et al., 2019). In contrast to traditional sports, the gaming community could not initially rely on an already developed system of clubs and associations. Instead, publishers created structures for this target group (eSport-Bund Deutschland e.V., 2018b; Scholz, 2019). Therefore, esports lack organizational and regulatory non-profit mechanisms omnipresent in traditional sports. A regulative and recognized governing body has yet to overcome game publishers' legitimacy and market-dominating position. Nevertheless, international (e.g., International Esports Federation, World ESports Association), continental (European Esports Federation), and national associations (e.g., Korean e-Sports Association, eSport-Bund Deutschland e.V., Japan Esports Union) have emerged in recent years.

Still, esports' prevailing regulatory principles remain unidentifiable (Peng et al., 2020). Scholz (2019) argued that these principles are unwritten but recognizable—even if not at first glance. Loose structures, publisher dominance, the self-image of the individual communities, and a large number of players create a metaphorical Wild West scenario. While in traditional sports, rules, regulations and systemic hierarchies constrained organizational activities, “the eSports industry, until now, has kept its start-up mentality” (Scholz, 2019, p. 111). This freedom initially shaped esports and provides countless opportunities to develop new and innovative ideas and structures. Abanazir (2018) claims that it is almost impossible to establish an umbrella organization for esports which regulates existing games, tournaments, and publishers. A standardized approach with a rigid governance model and the transfer of narratives from traditional sports is not well suited for esports (Peng et al., 2020). This is because “eSports is a collection of competitive gaming, and therefore not governable, as sports in their entirety are not manageable.” (Scholz, 2019, p. 111f.). A focus on a stakeholder-driven approach is necessary as governance is more fruitful in this context, which focuses on specific aspects of esports, such as individual teams or games (Kelly et al., 2022). However, esports is subject to the regulations of the respective publisher. This specific setting challenges traditional understandings of sport governance, particularly the role of associations in a profit-driven industry.

Given these intriguing developments and observations on the systemic governance of esports, in-depth research on the institutionalization of esports is highly relevant. Summerley (2020) provides initial approaches by examining similarities and differences in the institutionalization of traditional sports and esports. Most existing publications are preoccupied with debating whether or not esports are sports (van Hilvoorde and Pot, 2016; Funk et al., 2018; Hallmann and Giel, 2018). In this study, we consider esports as a real economic and, above all, social phenomenon and do not engage in its status as a sport. Thus, our study is an initial attempt to better understand the organizations behind the socioeconomic phenomenon of esports. Funk et al. (2018) and Heere (2018) claim that beyond rather descriptive observations on stakeholder interests and relationships, a theoretically sound analysis of institutionalization processes in esports is missing.

Scholz (2019) systematically categorized the different stakeholders in the esports ecosystem. He differentiates between primary (i.e., game developer, tournament organizer, professional teams and players, providers, and communities) and secondary stakeholders (i.e., governing bodies, sports organizations, sponsors, general public, investors, entrepreneurs, media, and shareholders). The multidimensional character of esports is also, and above all, reflected in the variety of genres into which the various game titles can be classified. Popular esports genres are first-person shooter games (e.g., Counter-Strike: Global Offensive), multiplayer online battle arenas (e.g., League of Legends and Defense of the Ancients II), real-time strategy games (e.g., StarCraft II), and sport simulations (e.g. FIFA) (Funk et al., 2018). See Besombes (2019) for a more nuanced overview of different genres and games. According to Hamari and Sjöblom (2017), esports operate in organized formats within various leagues and tournaments at the non-elite and or elite level. Esports events are watched by live, online, and broadcast audiences and can acquire millions of viewers (Funk et al., 2018). The esports ecosystem is subject to a constant change. These include new games, new genres, new tournaments and leagues, the emergence of new shareholders, as well as mergers and acquisitions. Nevertheless, esports multidimensional and dynamic character with different players and the multitude of existing games and genres is often neglected in previous research (Scholz, 2019).

To improve our understanding of esports' novel and complex governance, we focus on recent events in the German esports industry. The German esports industry is well developed and is one of the largest revenue-generating regions in the world (Deloitte Development LLC, 2020). In terms of esports penetration, Germany (33% in total; 7% occasional consumers, 11% regular consumers, 5% hardcore consumers) lags behind its direct neighbor Poland with a total of 52% (23% occasional consumers, 20% regular consumers, 9% hardcore consumers). Thus, Germany is only average when compared to other European countries, lagging behind esports strongholds such as Spain and Italy (Deloitte Development LLC, 2021a). Despite this, Germany is home to many elite esports players and unique top-tier competitions. The most important esports organization in Germany is the Electronic Sports League (ESL), headquartered in Cologne and host of major esports events such as the IEM Cologne. Unique tournament formats such as the Virtual Bundesliga and the regular season matches of the European League of Legends Championships (LEC) are based in Germany and attract large German companies as investors and sponsors (Deloitte Development LLC, 2021b). In addition to these organizations primarily focused on business and competition, Germany is also home to recently founded esports associations.

The first esports associations emerged in the first decade of the 2000s (Seo, 2013). Since 2016, different esports associations have been founded in Germany to represent stakeholders' interests. To develop professional esports, ESL founded the World ESports Association (WESA) in 2016 (World ESports Association, 2022). Whilst nominally an association, WESA is essentially the governing body of an esports league. As a reaction to the growing esports audience, the eSport-Bund Deutschland e.V.^2^ (ESBD) was initiated in 2017 as a non-profit association to govern esports in Germany. In 2022, ESBD has 67 members, mainly teams and clubs, as well as consultants, event organizers and content producers. ESBD is mainly focused on amateur athletes and teams (eSport-Bund Deutschland e.V., 2018c). ESBD has similarities with a traditional national sports-governing body (also known as a national sports organization or federation). The emergence of WESA and ESBD reflects the substantial growth and professionalization of esports in Germany. From a methodological perspective, the two newly founded associations provide a fruitful context for qualitative fieldwork on the institutionalization of associations within the esports ecosystem.

Against this backdrop, this study shifts the academic conversation on esports governance toward governing bodies. These organizations are all-powerful in traditional sports but live in the shadow of game developers in esports. We pursue the following research question: *How do German-based esports associations pursue legitimacy?* More specifically, we examine critically the legitimacy-seeking activities of WESA and ESBD.

## Theoretical Framework: The Concept Of Legitimacy

In line with neo-institutional theory, legitimacy provides the theoretical framework for our analysis. Early institutionalists (Meyer and Rowan, 1977; Zucker, 1977; DiMaggio and Powell, 1983; Meyer and Scott, 1983) observe substantial similarity among organizations operating in the same environment. This similarity results from structural and behavioral alignment to meet external pressure and accompany social expectations. Known as institutional isomorphism, this construct is crucial for organizations to secure legitimacy. Three mechanisms of institutional isomorphism can be distinguished: (1) coercive, (2) mimetic, and (3) normative (DiMaggio and Powell, 1983), even though the different types cannot always be empirically delineated. Instead, organizations do not adopt them one-to-one without situational adaptions. Organizations focus on individual case-specific solutions resulting from integrating new ideas and models into existing structures (Sahlin and Wedlin, 2013).

Legitimacy is closely linked to the cultural support that an organization can provide for its environment and audiences (Suchman, 1995). Ruef and Scott (1998) state that the degree of legitimacy depends on the evaluation of all involved stakeholders concerning different organizational aspects. These considerations are closely linked to where legitimacy comes from Deephouse and Suchman (2013) and for what it is used (Suchman, 1995). Both internal and external interest groups must be considered by organizations when pursuing legitimacy. This is most notably the result of the varying interests and views that stakeholders have concerning the legitimacy of an organization (Ruef and Scott, 1998). Stakeholders will only collaborate with legitimized organizations (Deephouse et al., 2017). Meyer and Rowan (1977) note that organizations with a lack of “acceptable legitimated accounts of their activities […] are more vulnerable to claims that they are negligent, irrational or unnecessary.” (p. 349 f.). According to Weber (1968), the importance of legitimacy lies in its ability to align organizational action with fundamental social values. An organization's formal structure (e.g., offices, departments, positions, and programs), is explicitly linked to its objectives, procedures, and policies (Meyer and Rowan, 1977). The evaluation of activities and the course of action (i.e., an organization's purposeful and goal-oriented work to meet individual and social values, norms, beliefs, and definitions) is socially constructed (Díez-Martín et al., 2021) and subjectively created due to different views of stakeholders. Suchman (1995, p. 574) wrote, “Legitimacy is a perception or assumption in that it represents a reaction of observers to the organization as they see it; thus, legitimacy is possessed objectively, yet created subjectively.” Therefore, the activities of an organization, aligned with the overall goals and the perception and evaluation by the respective stakeholders, are essential to ensure legitimacy.

Organizational legitimacy is “the perceived appropriateness of an organization to a social system in terms of rules, values, norms, and definitions” (Deephouse et al., 2017, p. 32). Suchman (1995) argued that an organization can proactively seek to gain, maintain, and recover legitimacy. Gaining legitimacy is essential for new entrepreneurial organizations (Aldrich and Fiol, 1994). Consequently, the organization has the task of identifying suitable actions that enhance its legitimacy in eyes of stakeholders (Ruef and Scott, 1998). A considerable body of research highlights how new organizations acquire legitimacy by conforming to existing norms and values (Meyer and Rowan, 1977; DiMaggio and Powell, 1983).

There is uncertainty about how new organizations can best acquire this legitimacy (Zimmerman and Zeitz, 2002; Bitektine, 2011). Suchman (1995) offers three different strategies for gaining legitimacy: (1) conform to existing environments and adapt preexisting environmental standards, (2) select among environments to ensure audience support, and (3) manipulate environments to promulgate new cultural beliefs. Zimmerman and Zeitz (2002) propose a further strategy: (4) creation of the environment. Table 1 summarizes the characteristics of the four strategies.

**Table 1:** Legitimacy strategies and characteristics.

**Strategy**	**Characteristics**	**Source**
Conformance	- Positioning in an existing institutional regime - Considering demands and expectations of existing structures or influential stakeholders - Align with already existing norms and rules	Meyer and Rowan, 1977; DiMaggio and Powell, 1983; Suchman, 1995; Mitchell et al., 1997; Zimmerman and Zeitz, 2002; Scherer et al., 2013
Selection	- Choice of a suitable and favorable geographical environment providing similar scripts, rules, norms, and values	Suchman, 1995; Zimmerman and Zeitz, 2002
Manipulation	- Counter existing cultural beliefs - Influencing social expectations using strategic instruments of public relations, e.g., lobbying or teaming up with already well-established organizations - Proactive promulgation of new destructive needs beneficial to the organization	Oliver, 1991; Suchman, 1995; Zimmerman and Zeitz, 2002; Scherer et al., 2013
Creation	- Developing new rules and regulations - Contradict social structures - Providing new scripts, rules, norms, values, and models for unprecedented new approaches	Aldrich and Fiol, 1994; Zimmerman and Zeitz, 2002

Legitimacy can be pursued using these strategies individually or in some combination. Legitimacy is confronted with measurement problems because “legitimacy is not directly observable” (Zimmerman and Zeitz, 2002, p. 418). Proposed measurement instruments “are not generalizable to other contexts, do not integrate the different approaches to assessing legitimacy, nor do they explain their suitability for specific contexts.” (Díez-Martín et al., 2021, p. 100). The multiplicity of measurement instruments increases scientists' uncertainty about which instrument is appropriate for each context. However, the lack of measurement is rooted in the subjectivity of the construct, which is exclusively limited to the attitudes and conscious and unconscious decisions of social actors. Measuring legitimacy is closely linked to evaluating organizational actions. Accordingly, some evaluations focus on specific groups of evaluators and measure legitimacy through media, customers, or regulators. Furthermore, different scales are applied to measure the construct. Other approaches measure legitimacy through linked typologies (Díez-Martín et al., 2021). Legitimacy researchers use quantitative content analysis (Deephouse, 1996; Ruef and Scott, 1998; Deephouse and Carter, 2005) or qualitative case studies combined with qualitative interviews (Rutherford and Buller, 2007; Low and Johnston, 2008; Goodstein and Velamuri, 2009).

## Materials and Methods

We examine the legitimacy-seeking strategies of two German-based esports associations: (1) the World ESports Association (WESA) and (2) the eSport-Bund Deutschland e.V. (ESBD). In line with the previous studies on legitimacy, we choose a qualitative case study. A case study approach is suitable given the reliance on qualitative data (Zimmerman and Zeitz, 2002). Yin (2018) suggests six potential sources of evidence for case studies. We rely on two sources of qualitative data: documents and semi-structured interviews.

### Documents

The documents analyzed in the study were website information, official press releases, and news retrieved from the associations' websites from 2016–2021 (WESA) and 2017–2021 (ESBD). 2016 and 2017 represent the founding years for the associations. The initial search generated 82 documents. As a first step, all available documents published by the associations since their foundation were read completely and screened to determine their relevance to the research question. In a second step, the documents were examined for indications of associations' efforts to acquire legitimacy. We removed reports on market data, news on market developments, and association personnel matters. Documents that provided information on new partnerships, strategies, goals, and activities were retained for further analysis. As the data are self-reported by the associations, the selected documents were discussed in detail by two authors in a final step. The final data set consisted of 55 documents. Table 2 shows the selected documents, separated by initial sample and final selected sources. The documents ranged in length from 200 to 3,000 words.

**Table 2:** Number of documents by initial and final sample.

**Organization**	**Number of documents**	
	**Initial sample**	**Final sample**
eSport-Bund Deutschland e.V.	53	38
World ESports Association	29	17
Total	82	55

### Semi-structured Interviews

Semi-structured, guideline-based interviews were also conducted *via* online video, audio-recorded, and then transcribed. The interviews provided a complementary data source to the documents. Interviewees had backgrounds in association work, across the amateur and professional esports spectrum. The initial intention was to include each stakeholder group by conducting one interview. As the most important stakeholder group, publishers were of particular interest in examining their view toward the emerging associational work in esports. However, our invitations were either unanswered or declined. Although publishers could not be included in our analysis, the ecosystem consists of many different stakeholder groups with a legitimate interest in esports associations. Notwithstanding this limitation, our sample of respondents still allows us to assess the legitimacy seeking strategies of both esports associations.

An interview guide was developed and structured according to four main topics: (1) development of the current model, (2) esports ecosystem and the publisher's dominant position, (3) perceptions of associations' legitimacy from a stakeholder's perspective, and (4) likely future developments. Subtle adjustments were made for each participant to reflect their organization's position in the esports ecosystem. Nine interviews were conducted between July and September 2019, each lasting between 26 and 53 min. Table 3 summarizes all interview information.

**Table 3:** Characteristics of participants and interviews.

**Interview**	**Participant**	**Description**	**Duration of interview (in min)**
1	Esports Athlete	Amateur esports player, former clan member (conducted *via* video)	00:48:10
2	Esports Athlete	Semi-professional esports player, active clan member and league player (conducted *via* video)	00:35:46
3	Esports Athlete	Semi-professional esports player, active clan member and league player (conducted *via* video)	00:44:39
4	Esports Athlete	Former professional esports athlete (Counter-Strike 1.6)	00:26:31
5	Professional Esports Team	Business Operations Manager esports	00:43:59
6	Professional Esports Team	Project Manager esports	00:49:48
7	Esports Event Organizer	Head of Public Relations	00:37:24
8	Esports Marketer	Sales Manager esports	00:40:48
9	Esports Association	Member of executive board	00:53:27

### Coding and Category Development

Data coding and category development for interviews and documents was conducted using MAXQDA12 software. A systematic and theory-guided approach to text analysis is mandatory to summarize the linguistic material and enable coding. Therefore, we followed the qualitative content analysis guidelines of Kuckartz (2014). Codes were developed according to the legitimacy strategies proposed by Zimmerman and Zeitz (2002). The underlying characteristics are presented in Table 1. Hence, the four strategies of (1) *conformance*, (2) *selection*, (3) *manipulation*, and (4) *creation* become the main categories for our analysis. Afterward, the research team reflected and discussed the relations between (sub-)categories and associations' legitimacy. The coding of the initial material was done in German. Citations used in this paper were translated into English. To avoid textual distortion due to the translation process, two authors fluent in both English and German (re-)translated the statements.

There is a potential risk for biases in data analysis due to the experience with the observed phenomenon by involved researchers (Berger, 2015). To limit this, a diverse research team was established. One research team member is an esports insider and, thus, close to the object of investigation. The other three researchers make no claims to insider status. The research team was also comprised of authors from different national and institutional backgrounds and academic career stages (two junior researchers and two senior researchers). The research team members also possessed expertise in a variety of scientific disciplines (i.e., organizational theory, management, economics, governance, and sociology). All of these ensured a multiperspective view on the phenomenon, enabled constructive bilateral and critical conversations among the research team, this ensuring reflexivity.

## Results

WESA and ESBD pursue different legitimacy seeking strategies. And not every strategy is equally relevant. Selection and creation are not as relevant as conformance and manipulation. Although the two organizations are located in German cities (i.e., the ESL as the founder of WESA in Cologne and ESBD in Berlin), a choice of geographical location as a strategy was not identified. Furthermore, creation is not applicable according to the initial definition (refer Table 1), although conformance and creation are difficult to separate. However, our results show more of a transfer or alignment with existing norms than creating new structures.

Each association will be analyzed individually to ensure a transparent and structured reporting of our results. The following similarities could be identified.

### Common Legitimacy Strategies

A legitimacy-seeking strategy shared by both WESA and ESBD is to transfer structures from traditional sports to esports. Results show a common orientation toward established associations in traditional sports as part of conformance as a strategy: “A traditional sports association has a great deal of know-how in many areas, which is also reflected in esports.” (Int_7; Esports Event Organizer). According to our analysis, this concerns different fields: the adaption of primary organizational forms, the proclamation of representing members' interests, and establishing tournaments and leagues. In addition, both organizations choose the organizational form of a registered non-profit association. WESA, as a more professional and international organization, is registered in Switzerland, as many international sports associations. ESBD is registered in Germany, similar to national sports associations. All the associations examined are formally constituted by a general statute, a purpose, and an executive board. Above all, the purpose is formulated with a clear non-profit orientation in both associations' statutes. While the statutes of ESBD are accessible on the association's website, WESA statutes are not accessible to the general public. We obtained WESA statutes from the commercial register in Switzerland.

The associations formulated goals to promote esports in general and create overarching standards. WESA focuses on regulating tournaments and leagues on a professional level. ESBD tries to cover talent development and setting standards mainly for amateur esports. Both consider themselves as responsible for dealing with their respective members and interests. This internal structure corresponds to that of most traditional sports associations.

“ESBD sees itself as an association of esports clubs and active players. […] I try to help strengthen esports' social position, make proven structures from traditional sports fruitful in esports, and bridge the gap between the classic world of sports and esports. There is a lot to learn from each other […].” (Int_9; Esports Association)

Again, both associations imitate strategies from traditional sports organizations, described by conformance as a strategy. One interviewee refers to WESA as the “champions league of esports” which “is also organized according to the classic methods of sports marketing and sports organization.” (Int_9; Esports Association)

Second, besides adapting established associational structures, we identified manipulation as another strategy pursued by both associations. More precisely, both associations created partnerships with well-established organizations. More details are presented separately for WESA and ESBD in the results section. The selection of partners is based on the objectives pursued. Partnerships are predominantly only established if they are advantageous for the targeted organizational environment of the association. WESA mainly focuses on commercial stakeholders. ESBD tries to improve connections to national and local governments and politicians as powerful actors in the German sports governance (Kurscheidt and Deitersen-Wieber, 2011). Conversely, other stakeholders do not consider associations as beneficial partners: “I do not think an association […] would help us at the moment if we would work with them.” (Int_6; Professional Esports Team).

The following two subsections will reflect each association by focusing on activities and strategies in the pursuit of legitimacy.

### World ESports Association (WESA)

WESA was founded in 2016 as “the result of joint efforts between industry-leading professional esports teams and ESL.” ESL is a German esports organizer and production company that produces video game competitions worldwide and is the self-proclaimed “world's largest esports company” (World ESports Association, 2022). The eight founding esports teams were Fnatic, Natus Vincere, EnVyUs, Virtus.pro, G2 Esports, FaZe, Mousesports, and Ninjas in Pyjamas. At its peak, there were 13 teams. The opening statement on the WESA website proclaims WESA as an “open and inclusive organization that will further professionalize esports by introducing elements of player representation, standardized regulations, and revenue sharing for teams.” (World ESports Association, 2022).

WESA's primary purpose is to serve the economic driven goals of the parent organization, ESL. Hence, the power of WESA teams is limited. Analyzing further cooperation with other profit-oriented and beneficial companies emphasizes this perception. WESA affiliated teams financially benefit from their membership and have representation on WESA's decision-making groups. According to one interview partner:

“WESA was then the first attempt to say, ‘Hey, we are forming a community with teams, and from now on, we will work together with the teams, who will then also have a veto in […] the supervisory board or in the committee.’ (Int_8; Esports Event Organizer)

Nevertheless, three out of six WESA board members are ESL representatives, ensuring ESL interests are protected and maximized. WESA is focused only on elite/professional esports. Non elite and or amateur esports is not a consideration. Professional teams are the only organizations affiliated to WESA. Publishers are best described as partner organizations, but not members or affiliated organizations of the league/association. The underlying concept is an economically oriented business model to achieve financial goals:

“WESA is a commercial institution that aims to bring together the world's best esports teams, bind them, and organize competitions on this platform […]. Thus, it is a commercial marketing platform, a commercial league structure/platform that is focused on making money […].” (Int_9; Esports Association)

Also important to WESA ecosystem are the streaming providers relevant because they broadcast and pay for the matches organized by ESL. The nature of esports also makes it necessary for WESA to partner with game publishers. The Pro League for *Counter-Strike: Global Offensive* (published by Valve Corporation) was established in 2016, and in 2017 for *Paladins: Champions of the Realm* (published by Hi-Rez Studios). However, neither publisher is a WESA member. In this context, one interviewee described WESA as a “Swiss army knife of league organization, hopefully attracting as many publishers and media partners as possible in the future. That is potentially a nine-digit million-dollar business.” (Int_9; Esports Association). Establishing networks to relevant stakeholders of the ecosystem belongs to the theoretical concept of manipulation.

WESA's ability to acquire legitimacy was impacted by decisions of certain esports teams to not affiliate. For example, leading non-German Counter-Strike teams such as Astralis, Vitality, or Team Liquid are not members. Domestically, also absent was Berlin International Gaming (BIG).

According to our data, WESA does not try to connect to other stakeholders outside the esports ecosystem. We found no evidence of lobbying local, national, or supranational governments or traditional sports organizations. WESA does not pursue manipulation strategies toward fans and viewers, although it explicitly addresses all participants in the esports ecosystem. These stakeholders are served indirectly as the focus of WESA is on “what interests the consumers” (Int_9; Esports Association).

In 2020, the ESL established the *Louvre Agreement*, which, amongst other things, excluded WESA as the league's governing body. Since the Louvre Agreement was announced, neither the news section of the website or its twitter feed have been updated. Even though WESA seems inactive, those in charge want to continue organizing other game titles under the association's umbrella (ESL Gaming GmbH, 2020; The Esports Observer, 2020).

Furthermore, at the beginning of 2021, Hi-Rez Studios—the developer and publisher of *Paladins*—withdrew its involvement in esports (including the Paladins Pro Circuit) to focus on improving *Paladins* as a game as distinct from esports. WESA did not publish any statement on this decision. Moreover, it is unknown to what extent WESA was involved in this decision. WESA's legitimacy was always limited given it was linked to only two game titles. However, the loss of *Paladins* exacerbated the situation.

Our analysis shows that WESA was a reliable and essential regulatory body for the target group. This created a platform for different stakeholders (Buser et al., 2022), including professional esports teams. The autonomy of WESA can indeed be doubted given it was founded by ESL. With the integration of commercial companies, WESA represents a closed system and, thus, ensures the economic success of ESL. The withdrawal of WESA as a regulatory body for the two ESL Pro leagues proves the organization's dependence on the ESL.

In summary, the legitimacy of WESA derives from both its members (professional esports teams) and the competitive league structures (in cooperation with the ESL and broadcasters like Facebook). Due to the Louvre Agreement, the organization lost the latter within a year. Teaming up with traditional sports is not considered. Furthermore, there is no political lobbying to meet the association's goals. Results suggest that the organization mainly uses manipulation as a legitimacy seeking strategy. Integrating professional esports teams into a closed, for-profit system is the main focus of WESA to build legitimacy.

However, a focus on specific stakeholder groups results in a lack of acceptance for the organization from the outside. Allegations of corruption due to their dependency on ESL will not enhance external stakeholders' perceptions of WESA's legitimacy. The lack of persuasion in publisher support for their activities indicates a further lack of recognition beyond their organization and members. The decision by Hi-Rez Studios to withdraw *Paladins* from the ESL Pro League reinforces this statement. As mentioned above, the statutes are not open to public inspection, which further contributes to a low level of transparency.

### ESport-Bund Deutschland e.V. (ESBD)

ESBD considers itself as the association responsible for organized esports in Germany. The aim is bring order to the fragmented German esports landscape. Our results show that ESBD is strongly oriented toward the structures of member associations from traditional sports: “ESBD is a traditional association. Just like sports associations.” (Int_9; Esports Association). Accordingly, conformance as a strategy to gain legitimacy can be observed, as they imitate structures from legitimate sports associations. The alignment with internal structures of traditional sports associations has already been mentioned above.

ESBD is focused on implementing the pyramidal-hierarchical structure evident within the traditional sports system. (Self-)organized amateur esports form the basis of this system, but ESBD has also sought links with professional teams: “Yes, they have already been here. We talked to them once, but they are more traditional in terms of grassroots sports.” (Int_6; Professional Esports Team). According to our interview partner, no further cooperation was established, because ESBD could not offer anything to the professional team.

Since 2019, ESBD established an amateur league for association-registered grassroots teams. In establishing the league, ESBD sought a broad and unique competition structure for amateur and non-elite players. ESBD wanted to make self-organized competitions redundant, by providing a transparent and credible league system. Amateur teams compete in four disciplines: *Counter-Strike: Global Offensive, StarCraft II, Rocket League*, and *League of Legends*. As an ESBD member, the ESL acts as the league's organizer. In traditional German sports, the associations organize competitions and set their rules with international federations (Chappelet, 2010). ESBD leagues are not connected to professional leagues and neither do they attract all amateur teams. Therefore, setting up an own league for amateur clubs is a kind of mimicry related to the strategy of conformance and not creation, as they are not successfully created structures or rules.

ESBD seeks to have a cooperative relationship with the traditional sports system:

“Overall, we are striving for a collaborative relationship with traditional sports and its structures in the short and medium-term: the mutual exchange of expertise and experience is independent of any possible organizational integration into the organized sport and can be expanded through concrete cooperation and joint projects.” (eSport-Bund Deutschland e.V., 2018b).

The activities in this area are many and varied. For example, ESBD supports traditional sports clubs that have integrated esports as a separate division. The main goal of this cooperation is to transfer traditional sports organizational knowledge to esports, for example, standards of training organization or the integration of voluntary work.

“The goal of these cooperations is to actively shape the integration of esports into sports society, to transfer sports organizational knowledge from the traditional area e.g., training design, integrity assurance, volunteer organization, and to make this knowledge available for the continuous further development of esports—both in terms of sports and society.” (eSport-Bund Deutschland e.V., 2018b).

Besides conformance, ESBD uses manipulation in the pursuit of legitimacy by focusing on partnerships *across* esports. Partnering with successful and well-established organizations generate a higher impact and growth (Zimmerman and Zeitz, 2002). Alliances with professional teams (e.g., Berlin International Gaming, Unicorns of Love), event organizers (e.g., ESL, Freaks 4U Gaming), and amateur esports clubs (e.g., Leipzig eSports e.V., Magdeburg eSports e.V.) are part of their network to generate potential synergies. Membership is granted upon application. Applicants must either be an organizer of an esports gaming operation or actively participate in such.

In another legitimacy seeking action, ESBD sought and became affiliated with the newly founded European Esports Federation, as well as the International Esports Federation. As one interviewee noted, ESBD “took the leadership” toward this development (Int_9; Esports Association). This is a strategy of conformance, as it copies the hierarchical structures of traditional sports.

In terms of lobbying and public relations work, ESBD tries to strengthen its position. According to our results, politics plays an essential role in this context. ESBD initially focused on regional and national political institutions as well as essential decision-makers to generate an understanding of esports and its essential functions and structures among the general public:

“The social acceptance for esports exists, and it is strong. We now want to achieve a sustainable and deep integration of esports. To this end, we will initially accompany politics in particular on this core topic.” (eSport-Bund Deutschland e.V., 2018a).

In particular, press releases and news items suggest that the most critical issue in cooperation with political decision-makers is the recognition of esports as a sports activity. ESBD's involvement in political events and debates are content of these published items. This goal has been pursued vigorously since the associations' foundation until today without only modest success. ESBD proclaimed a small victory, when in 2019, German immigration law was revised to provide esports athletes with the same visa and travel requirements as elite athletes. WESA also participated in this lobbying campaign, motivated by more streamlined processes to bring non-German esports athletes to Germany to play in events. This concession raises esports, at least in this respect, to the same level as other sports—one of ESBDs main approaches for legitimacy. The press statement concerning this topic expressed ESBD's pursuit for legitimacy. ESBD emphasize the importance of their success, even if the effect is limited, especially for an amateur organization. However, they proclaim: “The visa issue has blocked the development of the German esports landscape for years.” (eSport-Bund Deutschland e.V., 2019).

In summary, our results show that emerging esports associations in Germany use conformance and manipulation as major legitimacy strategies. This is manifested by aligning their associational structures to those evident in traditional sports organizations and the implementation of beneficial networks to enhance stakeholder perceptions of legitimacy. Both interviews and documents provided equal evidence of the two associations' approaches. Table 4 provides an overview of the complementary use of the two sources and an excerpt of additional exemplary statements that highlight conformance and manipulation strategies used by the associations.

**Table 4:** Selected exemplary interview statements and documents citations.

**Strategy and characteristics**	**Source**	
	**Interviews**	**Documents**
**Conformance** - Positioning in an existing institutional regime - Considering demands and expectations of existing structures or influential stakeholders - Align with already existing norms and rules	[…] As I said, the way they build up the whole casting around esports is one hundred percent copied from traditional sports or partly improved from those areas, and therefore it all looks very professional at the moment. (Int_8; Eports Marketer) Establish a structure that ensures that everyone deals with each other in a cultured and decent manner. That is what a traditional sports association wants to do—it also wants to ensure we are healthier and good taxpayers. That is what an esports association also strives for […]. (Int_9; Esports Association) We co-founded ESBD, have a permanent seat on its board, and are trying to work together with the teams, both amateur and professional, to professionalize esports in Germany, to further strengthen it, and to establish guidelines. In other words, to create standards so that esports in Germany can continue to grow. (Int_7; Esports Event Organizer)	As is familiar from traditional sports, there is also a pyramid-like organization in esports: the basis, the foundation, is formed by the players, who deal with the esports titles individually, on gaming platforms and networks, often online, and enter into the active gameplay (eSport-Bund Deutschland e.V., 2018b) Based on similar traditional sports associations, WESA is an open and inclusive organization that will further professionalize esports by introducing elements of player representation, standardized regulations, and revenue shares for teams (World ESports Association, 2022) The eSport-Bund Deutschland (ESBD) has continuously promoted easier entry conditions for esport athletes and already succeeded in implementing short-term visas last year (eSport-Bund Deutschland e.V., 2020)
**Manipulation** - Counter existing cultural beliefs - Influencing social expectations using strategic instruments of public relations, e.g., lobbying or teaming up with already well-established organizations - Proactive promulgation of new destructive needs beneficial to the organization	[…] politics must develop an understanding […] of what esports is, the needs of those who practice it, the barriers, and the needs that politics must also address. (Int_9; Esports Association) Yes, and then they would have to start [...] to become active. Sitting in the VIP area is, I think, quite lovely, but it does not help the community because you cannot get to know them, you cannot have controversial discussions. (Int_3; Esports Athlete) Moreover, WESA was then the first attempt to say: Hey, we are forming a community along with the teams and working together with the teams from now on, who will also have a veto in the [...] supervisory board or the committee. (Int_7; Esports Event Organizer)	The state government is asked to support and accompany the dialog between esports and traditional sports, including the recognition of esports as an eligible sport within the meaning of § 2 No. 1 of the statutes of the Landessportbund—while respecting the autonomy of the sport (Landtag von Sachsen-Anhalt, 2018) WESA will aim to incorporate more Teams and leagues, and will always work very closely with game publishers to include more games in the future (World ESports Association, 2022) YouTube will be the new streaming partner for Pro League Seasons 5 and 6 and will exclusively stream the English-language broadcast (World ESports Association, 2017)

## Discussion

This study sought to identifying the legitimacy strategies pursued by German-based esports associations. The remainder of this discussion is divided into three subsections. In the first two sections, conformance, and manipulation are discussed as legitimacy-creating strategies. The third section discusses the stakeholder-oriented approach of associations, which offers them a unique proposition in the fragmented esports landscape. Constructs from the scientific literature support our reasoning.

### Does Institutional Isomorphism Always Legitimize?

In traditional sports, there are independent, self-regulating, and non-profit-oriented (global) organizations that are well accepted as the legitimate governing body for each sports (Croci and Forster, 2004; Chappelet, 2010). It is therefore unsurprising that new esports associations start to exhibit the same characteristics of these organizations. Both WESA and ESBD use conformance to establish a transparent and basic structure for their organization. The associations examined are characterized by their constitution with statutes, articles, and standard binding and longer-term goals, varying to represent members' interests. The establishment and support of leagues are inherent to associations from traditional sports. The associations' approach reflects mimetic isomorphism (DiMaggio and Powell, 1983). Uncertainty about the future and the uncertain survival of an organization encourages it to align with dominant organizations and their structure and actions: “When goals are ambiguous, or when the environment creates symbolic uncertainty, organizations may model themselves on other organizations.” (DiMaggio and Powell, 1983, p. 151). This isomorphism usually achieves a taken-for-grantedness that finally secures legitimacy.

Our results suggest that the transfer of structures from traditional sports does not necessarily ensure legitimacy. This refers mainly to associational work in an esports ecosystem dominated by game publishers. Legitimacy—a resource nearly always evident in the associations responsible for traditional sports—cannot be acquired by esports associations through simple imitation. Even if the associations orientate toward structures from traditional sports, this does not simultaneously mean an increase in legitimacy as a simple transfer is not practicable (Kelly et al., 2022). Thus, a rigid institutional isomorphism is not likely to be successful. Case-specific and the nuanced use of imitation, which reflect the particularities of esports, will likely generate superior outcomes (Sahlin and Wedlin, 2013).

### Manipulation—A Fragile Bubble

Besides conformance, we identified manipulation as a strategy used by esports associations in the pursuit of legitimacy. Manipulation goes beyond pure conformity and environmental selection as organizations promulgate their distinctive needs and new approaches to operating cultural environments (Ashforth and Gibbs, 1990; Aldrich and Fiol, 1994). Our results show that the success of a manipulation strategy depends on the fundamental orientation of associations. In particular, ESBD uses the strategy to achieve its goals. While both associations build a network of profitable partnerships, only ESBD engages in political lobbying. The news items published underline this approach and, thus, highlight the associations' policy-oriented PR strategy to justify their requirements. Therefore, the proof of legitimacy is provided by constant demands on politicians since the associations must initiate consensual actions due to the various interest groups involved. Decision-makers, partners, and members are carefully selected and addressed. WESA is exceptional in this context as its monopolistic network was formed around specific game titles without the need for government support.

Partnerships and lobbying are inevitably accompanied increased dependence. According to our observations, most objectives can only be reached and implemented *by* or *through* governmental involvement. Even though politics is not integrated into WESA's approach, the association was critically dependent on the support of a single stakeholder, ESL.

The effectiveness of the manipulation approach to gain legitimacy is speculative. If the associations are not restricted in their objectives, nothing can be said against linking their work with their partners in politics and business. The dependencies raise concerns about the long-term sustainability of the cooperation. Because of a missed target achievement, even involved stakeholders might doubt the legitimacy of the respective associations. Conversely, this also means a loss of legitimacy for the association as “such proactive cultural manipulation is less controllable, less common, and, consequently, far less understood than either conformity or environment selection.” (Suchman, 1995, p. 591). We would even argue that the activities pursued to implement the manipulation strategy are *fragile bubbles* that threaten to burst at the slightest setback.

### Differentiation by Creating Stakeholder-Related Legitimacy

Organizations without legitimized activities are often viewed as unnecessary by their respective stakeholders (Meyer and Rowan, 1977). Therefore, the organization's legitimacy is closely linked to how stakeholders evaluate these activities and the added value they generate. Due to the diversity of esports with different games and genres, it is almost impossible to bundle all stakeholders in one overarching umbrella organization (Abanazir, 2018).

“So, you definitely need some kind of USP. On the one hand, we have WESA, which concentrates on a single game title. That is one way. On the other side, we have ESBD focused on a certain regional USP. You must limit yourself because esports, in general, is so complex and big with all the publishers, games, and agents […]. It is hardly feasible to provide an all-encompassing association.” (Int_7; Esports Event Organizer).

According to our findings, associations have recognized the need for such differentiation and focus on unique goals and activities. In addition, their actions are targeted to specific stakeholders to develop their own unique (sales) propositions. We are convinced that this differentiated approach can be successful in practice. Given the fragmented esports landscape, associations in esports need to focus on selected sub-areas, genres, or disciplines. This focus creates an orderly environment for stakeholders in a disorderly novel phenomenon and generates stakeholder-related legitimacy, which is a fruitful approach, according to Kelly et al. (2022). These bodies “must implement their own governance strategies and seek to legitimize those strategies in the eyes of relevant stakeholders.” (Kelly et al., 2022, p. 154). Despite serving different stakeholders, associations are also increasingly corporate to achieve common overarching goals, as demonstrated by the call for uniform visa standards, the development of beneficial partnerships, or the linkages of ESBD to other associations at the international level. Therefore, our results also confirm the assumption of Peng et al. (2020, p. 11) as “although struggling with legitimacy issues, new esports governance alliances are following a trend of moving away from fragmentation to a network administration organization (NAO) model.” Such a model offers the possibility to bundle common interests and enables a strategic approach in line with the overall network goals. The mentioned legitimacy problems have been highlighted in more detail in this study and the strategies used by associations to counter them. Although we cannot evaluate the intensity of cooperation, a tendency toward cooperation with as many other partners as possible to build up profitable networks is evident.

Due to their short existence, no statements can be made about the extent to which associations can establish themselves in the future as legitimate and recognized organizations while focusing on unique approaches. The degradation of WESA and its inactivity since 2020 indicates typical issues an association has to deal with in the emerging and dynamic field of esports: the dependency on (political) partners and stakeholders and, overall, a lack of publisher support for their activities. In addition, the question of the general need for associations in esports remains, accompanied by a lack of support from external stakeholders and the general public. Therefore, the associational work in esports is stuck in the middle, somewhere between publisher dominance on the one hand and the striving for independent structures on the other hand. Whether the assumed legitimation strategies of conformance and manipulation will be sufficient to solve these issues in the future can only be speculated at this point.

The pioneering work of associations has already initiated essential steps toward the future of esports. We can determine that the associations promote growth and raise awareness of their work. These actions try to create a certain level of order in a previously fragmented esports landscape with loose clans, confusing competitive structures, and a lack of responsibilities for target groups.

## Conclusion And Implications

Our study contributes to a better understanding of how (for-profit) leagues and self-proclaimed (non-profit) national governing bodies pursue legitimacy. In the context of limited academic discussion of governance in esports, our results generate preliminary but important managerial, policy, and theoretical implications.

### Management Implications

Our findings have implications for management to help associations further consider and rethink their strategic direction. A simple transfer of governance structures from traditional sports to esports is unsuitable (Kelly et al., 2022). Associations need to be more selective and find unique and targeted approaches rather than strive for an esports' all-encompassing governance solution (Sahlin and Wedlin, 2013). Thus, the associations have the chance to fill niches that remain unoccupied by publishers due to their profit-oriented focus. These blind spots (Peng et al., 2020) can be addressed with innovative approaches and individual target group-oriented solutions like new competitive structures (e.g., WESA) or a focus on amateur esports (e.g., ESBD). By filling the niches, associations reach individual stakeholders, creating stakeholder-driven legitimacy. The associations' work and orientation thus serve not only as a service for stakeholders but also for publishers, enabling them to focus on their core business, the distribution and marketing of their games. Associations need to accept publisher dominance. Their dominance is not challengeable. Instead, collaborative dialogue and exchange with publishers may establish a mutual understanding and enhance each other's legitimacy. The associations are operating in a highly competitive environment. Various stakeholders are striving for a position in this financially lucrative ecosystem. Esports associations must think more economically and entrepreneurially than associations in traditional sports. Focusing on specific stakeholders creates a space where publishers and other acting stakeholders can cooperate, benefit from each other, and coexist in a fragmented environment.

### Policy Implications

The institutional development of esports is barely comparable to those of long-established traditional sports. The fragmented environment with different games, genres, and stakeholders and the regulating power of the publishers characterizes esports unique structure (Scholz, 2019). Esports associations operate in a highly profit-oriented environment in which they must constantly prove their raison d'être, especially their economic value to commercial organizations. In contrast to traditional sports, esports associations cannot rely on a taken-for-granted legitimacy (Croci and Forster, 2004). The legitimacy of esports associations must be earned. In this context, we consider esports to be a blueprint for many subsequent sports that are confronted with comparably fragmented and developing governance structures. Our insights and the observed narratives have therefore policy implications for emerging sports, such as boardsports (Strittmatter et al., 2019). Esports is a growing ecosystem with rapid developments during the last decades. Governing organizations did not develop to the same extent. Like other emerging sports, institutionalized rules and organizations must be established over time.

### Theoretical Implications

Our research finally contributes to the organizational theory literature on associations in a profit-driven environment by identifying possible strategies these organizations use to pursue legitimacy. Suchman (1995) and Zimmerman and Zeitz (2002) propose four strategies: conformance, manipulation, selection, and creation. According to our findings and related to our case, conformance and manipulation are relevant strategies for esports associations to gain legitimacy. However, in the esports context, this legitimacy is often stakeholder-related and arises from focusing on individual stakeholder groups in a fragmented ecosystem where all-encompassing governance is not appropriate (Abanazir, 2018; Peng et al., 2020). More generally, the ongoing legitimacy discussion on esports governance is actually caused by the representatives of the esports ecosystem themself due to a conceptual and cultural annexation of the sport concept. The term “esports” per se implies a connection to traditional sports and the associated structures although the basic meaning just refers to the competitive, sports-like mode how video games are played. The accompanying expectations regarding a need for regulation and the associated *commitment to institutionalization* are the inevitable result. In the esports industry, however, *partial legitimacy* prevails, unevenly distributed among the relevant actors and their associated interests and rights in the ecosystem. Following our policy implications, which apply to comparable governance challenges in many new and emerging sports (e.g., Strittmatter et al., 2019), we recommend considering this novel construct of a partial legitimacy in the field of organization sociology and specifically in institutional theory. We recognize this as a gap in the literature and a need to extend theories on legitimacy creation.

### Limitations and Future Research

The present study provides valuable insights into legitimacy strategies esports associations pursue to ensure their survival and discusses associational work in a profit-driven business. However, several shortcomings must be pointed out, providing a basis for future research.

Despite nine interviews with esports stakeholders, it would be particularly desirable to include publishers in future research. It would be insightful to learn how publishers perceive associations. Hence, future research should examine the relationship between associations and publishers in more detail to uncover possible linkages. This supports the approach proposed in the management implications. A further limitation relates to the selected documents, representing a favorable perspective on the associations' work as we focused on primary documents published by the associations themselves. Accordingly, not all internal and external debates are depicted. The official statements only hint at the debates but limit us to a further interpretation. At the same time, this approach limits the number of fruitful documents to those published and approved by the associations. Nevertheless, the selected documents as primary sources provide valuable and meaningful insights into the two associations' actions which was the major purpose of this study.

This article focuses primarily on how associations use strategies to gain legitimacy in a first step. However, this is not sufficient for an organization to ensure its survival. In addition to gaining it, *maintaining* the achieved legitimacy and the ability to *repair* it in an unforeseen crisis are further challenges for organizations. For this purpose, different strategies are suggested in the literature (e.g., Suchman, 1995), which should be investigated. We moreover acknowledge that the legitimacy strategies are not always clearly distinguishable. In particular, conformance and creation are difficult to separate. Observed activities such as establishing uniform visa standards for esports athletes, and the foundation of leagues are possible creation approaches. In our interpretation, neither activity creates unique and new structures. Instead, they adapt structures from traditional sports, although similar league formats have not previously existed in esports.

In addition, the intercultural and international transferability of findings obtained in our study may be restricted. An association's legitimacy is likely to be country-dependent. Also crucial in this context is the general acceptance of esports in this respective environment. A recognition facilitates the associations' work and enables them to benefit from various resources from traditional sports (e.g., tax benefits or subsidies). Thus, the transferability of the results can only be guaranteed by extending the study to other countries.

As previously mentioned, the community has a historically shaped self-image in esports, as many statements confirm in our interviews. Donnelly (2013) argues that the democratization of sport by involving fans and players offers a variety of potential outcomes for the further development of a sport. Hence, the community's perception and attitude toward different governance models (with, e.g., a national, global, game- and/or team-based focus) require further examination, for instance, by larger-scale interview and survey methods or novel approaches for digitalized social environments, such as netnography.

Finally, we stress the complexity of esports. Our review of the literature has shown that complexity of the esports ecosystem has not been addressed adequately. Perhaps esports needs to be generalized and developed as a distinct research field to provide a holistic picture of the esports landscape. This includes associational structures, various game titles and genres, the different communities, the game publishers, and many other aspects and actors. We expect that academic interest in esports will continue to grow.

## Data Availability Statement

The raw data supporting the conclusions of this article will be made available by the authors, without undue reservation.

## Author Contributions

HH and MK were responsible for the conceptualization and design of the study. HH and CB were involved in the collection and analysis of the data. All authors listed contributed to the study, manuscript development, and approved the submitted version.

## Funding

This work was funded by the Deutsche Forschungsgemeinschaft (DFG, German Research Foundation) - 491183248 and Open Access Publishing Fund of the University of Bayreuth.

## Conflict of Interest

The authors declare that the research was conducted in the absence of any commercial or financial relationships that could be construed as a potential conflict of interest.

## Publisher's Note

All claims expressed in this article are solely those of the authors and do not necessarily represent those of their affiliated organizations, or those of the publisher, the editors and the reviewers. Any product that may be evaluated in this article, or claim that may be made by its manufacturer, is not guaranteed or endorsed by the publisher.

## Footnotes

^1^We use the term *publisher* throughout for the owners of the property rights of the games. However, publishers and game developers are not always the same corporations and the division of rights may differ.

^2^e.V. (eingetragener Verein) designates a registered non-profit organization in Germany, benefitting from tax exemptions.
